# Age and Species Comparisons of Visual Mental Manipulation Ability as Evidence for its Development and Evolution

**DOI:** 10.1038/s41598-020-64666-1

**Published:** 2020-05-06

**Authors:** Hrag Pailian, Susan E. Carey, Justin Halberda, Irene M. Pepperberg

**Affiliations:** 1 Harvard University, Cambridge, USA; 2 Johns Hopkins University, Baltimore, USA

**Keywords:** Evolution, Human behaviour

## Abstract

Intelligent behavior is shaped by the abilities to store and manipulate information in visual working memory. Although humans and various non-human animals demonstrate similar storage capacities, the evolution of manipulation ability remains relatively unspecified. To what extent are manipulation limits unique to humans versus shared across species? Here, we compare behavioral signatures of manipulation ability demonstrated by human adults and 6-to-8-year-old children with that of an animal separated from humans by over 300 million years of evolution: a Grey parrot (*Psittacus erithacus)*. All groups of participants completed a variant of the “Shell Game”, which required mentally updating the locations of varying set sizes of occluded objects that swapped places a number of times. The parrot not only demonstrated above-chance performance, but also outperformed children across all conditions. Indeed, the parrot’s accuracy was comparable to (and slightly better than) human adults’ over 12/14 set-size/number-of-swaps combinations, until four items were manipulated with 3–4 swaps, where performance decreased toward that of 6- to 8-year-olds. These results suggest that manipulation of visual working memory representations is an evolutionarily ancient ability. An important next step in this research program is establishing variability across species, and identifying the evolutionary origins (analogous or homologous) of manipulation mechanisms.

## Introduction

We live in a dynamic world, where objects move across time and space, disappear from our line of sight, and undergo featural transformations. To flexibly adapt to our changing environments, we invoke the visual working memory (VWM) system to perform two essential functions^1^. First, *storage* processes enable us to create mental representations of objects when they are no longer directly perceived. Second, *manipulation* operations allow us to alter these representations, by updating them upon encountering novel information or mentally simulating various events. Taken together, VWM storage and manipulation provide a means to reason beyond our perceptual experiences, giving rise to intelligent behavior.

Despite its functional significance, the VWM system is severely constrained^1^. Behavioral and neurophysiological work have identified signature limits in human storage capacity. On average, individuals are able to store a maximum of 3–4 items simultaneously^2–9^. Similar behavioral limits have been observed in other animals (e.g. birds and primates), suggesting functionally comparable brain networks to those supporting human storage^10–12^. Such findings shed light onto the evolution of VWM capacities, pointing towards possibly homologous mechanisms and shared limits underlying storage ability.

Much emphasis has been placed on delineating the origins of this storage capacity, with less focus placed on characterizing limits in VWM manipulation. However, manipulation constitutes the operative “working” component of the system that facilitates a variety of broader cognitive functions (e.g. comprehension, STEM aptitude, abstract/creative thinking)^1^. Moreover, the evolutionary history of this function has largely been unexamined, as existing behavioral research has lacked paradigms necessary to engage and test non-human animals^10^. Is mental manipulation a uniquely human ability, or can comparable competence be observed in non-human animals? Identifying (dis)similar manipulation capacities along the phylogenetic tree may provide insights into how evolutionary ancient this cognitive function might be, the environmental pressures by which it was engendered, and the neurocognitive mechanisms required to support it. Given the central role that VWM manipulation plays in supporting complex cognition, examining the evolutionary history of this function may also provide insights into understanding factors that may differentiate human intelligence from that of other species.

Here, we compare VWM manipulation capacity of a Grey parrot (Griffin) with that of human adults (n = 21) and 6-to-8- year old children (n = 21). These groups are well-suited for our present purposes, based on the following rationale. First, we presumed that Grey parrots likely possess some capacity to manipulate visual information, given environmental pressures placed on these birds in the wild (e.g. tracking location of moving fledglings, predators, etc.) and a neural architecture functionally homologous to those supporting human memory^10,11,13,14^. Thus, similarities or differences in memory manipulation ability between the parrot and humans may provide insights into the comparative evolution of this ability in the 300 million years since their last common ancestor^15,16^. Second, previous participation in experiments not directly testing visual working memory present Griffin as a particularly well-suited subject for prolonged experimental testing^17^. Third, baseline differences between Griffin’s storage ability and those observed in humans would require multiple groups for comparison. If Griffin is unable to store information equally as well as human adults, it would be unclear whether any manipulation-related differences actually reflect differences in manipulation capacity or initial storage ability (as information must first be successfully stored for it to be subsequently manipulated). As such, we included 6-to-8-year old children as a comparison group, given work establishing that storage capacity does not reach adult-like levels until beyond this age range^18–20^ and that Griffin has previously demonstrated comparable cognitive capacities to 6-to-8 year-olds on various tasks, quite likely as a consequence of his extensively documented training in verbal symbolic representation^21–24^.

**Figure 1:**
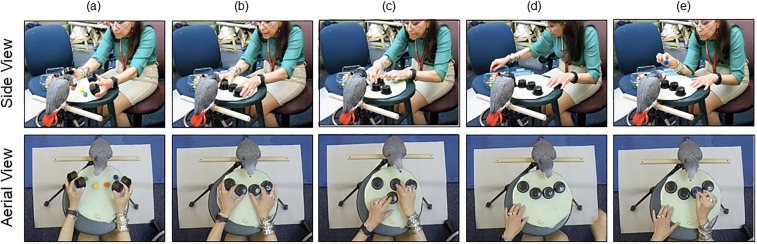
(**a**) Memory Period: colored stimuli presented; (**b**) Retention Period: all objects occluded, requiring color-location information to be stored in memory; (**c**) Swaps: pairs of occluded items swap positions *n* times, requiring color- information bindings be updated; (**d**) Reconsolidation Period: All objects remain stationary; (**e**) Test period: Subject is shown the targeted color and must indicate (peck or point) to the position where that color is expected.

We adapted the “Shell Game”^25^ to identify behavioral limits in a case study of VWM manipulation: the updating of color-location bindings. This task proved intuitive and engaging to the extent that we were able to test different species and human age groups using the exact same methods. In this live experiment (Fig. 1), the Grey parrot, human adults, and 6- to 8-year-old human children were briefly presented with varying set sizes (2–4) of colored objects (woolen pompoms) that were subsequently occluded by opaque cups. On some trials, the cups remained stationary (0 swaps), requiring participants to store mental representations of these objects in memory. However, for most trials, pairs of cups swapped positions multiple times (1–4 swaps), requiring participants to manipulate their mental representations by updating the color-location information of the moving objects (i.e. which pompom went where). After all swaps were complete, participants were shown a target colored pompom and instructed to indicate the cup under which they expected to find the object that matched that color. Notably, the parrot did not receive special training relative to humans, as it was able to grasp task pragmatics by simply observing the experimenter and a confederate perform three example trials. The parrot had already demonstrated full Stage 6 Piagetian object permanence:^26^ a basic ability to track invisible displacements and succeed on a simple version of the standard Shell Game (tracking the movement of one object hidden under one of three cups). To ensure that the 6-to-8-year olds remained cooperative and attentive during the experiment, the duration of children’s testing session was shortened by reducing the number of trials per condition and eliminating 4 swap trials (longest in duration) altogether.

We first present data observed in human participants, as we subsequently use these to benchmark the parrot’s manipulation ability. With respect to storage capacity, adults’ performance on no-movement (0 swap) trials replicated previous work demonstrating a 3–4 item storage limit [set size 2 vs. 3: t(20) = −1.00, p = 0.33; set size 3 vs. 4: t(20) = 3.16, p < 0.01]. Average performance on these trials exceeded 96% accuracy for all set sizes. Examining mental manipulation capacity, a 3 (set size: 2–4) by 5 (number of swaps: 0–4) repeated measures ANOVA performed on adult data yielded a main effect of set size, F(2,40) = 41.48, p < 0.001. Post-hoc contrasts revealed that performance was highest for conditions containing 2 items [set size 2 vs. 3: F(1,40) = 20.30, p < 0.05] and lowest for those containing 4 items [set size 3 vs. 4: F(1,40) = 17.50, p < 0.05]. The ANOVA also produced a main effect of number of swaps, F(4,80) = 20.33, p < 0.001. Performance on no-movement trials was higher relative to those containing swaps, F(1,80) = 53.44, p < 0.05, suggesting that some costs are indeed associated with additionally manipulating information in VWM. Performance accuracy decreased linearly as a function of the number of swaps that were performed, F(1,80) = 45.37, p < 0.05. A significant interaction of set size by number of swaps, F(8,160) = 6.83, p < 0.001, revealed that the magnitude of these manipulation costs were exacerbated by the number of items held in memory. Namely, the decrease in performance accuracy associated with manipulating 2 items across multiple swaps was significantly lower than that in trials in which 3 or 4 items were manipulated, F(1,160) = 32.53, p < 0.05. Results from a computerized variant of this task (see supplementary materials) suggests that this pattern cannot be attributed to tracking strategies or temporal decay. Moreover, given that adults were asked to verbally articulate two-digits aloud during each trial, it is unlikely that performance reflects a verbal rehearsal strategy^27,28^. Taken together, these findings reveal a reliable signature of adult mental manipulation ability observed in the Shell Game.

To investigate developmental differences in VWM, we first analyzed storage capacity. Planned comparisons on 0 swap trials for larger set sizes revealed that children performed significantly worse compared to adults at Set Size 3, t(40) = 2.83, p < 0.01, and Set Size 4, t(40) = 2.09, p = 0.02. These results replicate previous work demonstrating that VWM storage capacity does not reach adult levels until beyond age 8^18–20^. Turning to manipulation capacity, we conducted a three-factor mixed ANOVA, comparing performance across groups (6-to-8-year-old children vs. adults) on repeated measures of set size (2–4) and number of swaps (0–3). This analysis yielded a significant interaction of set size by group, F(2,80) = 4.96, p < 0.01. Although the ANOVA did reveal a main effect of number of swaps, F(3,120) = 17.59, p < 0.001, the interaction between age group and set size was merely trending; F(3,120) = 2.40, p = 0.08; see Supplementary Table 1 for full report of the ANOVA’s. Across both age groups, as with adults alone, performance decreased linearly as a function of the number of swaps, F(1,120) = 62.62, p < 0.05. The overall magnitude of these manipulation costs were exacerbated by set size, F(6,240) = 4.14, p = 0.001. However, the three-way interaction of set size, number of swaps, and groups was not significant, F(6,240) = 0.42, p = 0.81, so there is no evidence that the interaction between set size and number of swaps differs between adults and children. Both children and adults were able to manipulate 2 items across multiple swaps with little-to-no cost, relative to larger set sizes, F(1,240) = 24.90, p < 0.05. The lack of interactions involving age group on the effects of number of swaps should be viewed with some caution, given than children were not tested on four swap trials; these results nonetheless suggest that separate developmental trajectories exist for storage vs. manipulation, and should prompt further work investigating whether these abilities rely upon shared versus separate resources^25^.

**Figure 2:**
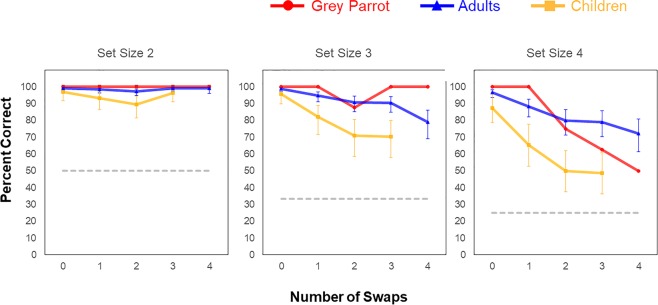
Model estimates of accuracy (percent correct) for each combination of set size by number of swaps, for each group of participants. Dashed grey line represent chance performance for each condition.

The above results set the stage for our main question: Are these manipulation abilities unique to humans, or are they manifest in the Grey parrot?” Bootstrap analyses (see supplementary materials) show that the parrot performed above chance-level across all conditions. However, given practical and resource limitations, our parrot sample was restricted to a single participant and we were unable to use frequentist statistical analyses to perform cross-species comparisons (as this would violate standard assumptions of independence). For this reason, we developed a linear mixed-effects model based on children’s and adults’ data. This analysis established predicted performance of each age group at each set size/number of swaps trial type with confidence level of 95% that the true group performance fell between a modeled low point and modeled high point. The model’s data, with the confidence level indicated by error bars, are shown on Fig. 2. We then conducted a point estimate analysis, where we compared the parrot’s performance to humans’ by determining whether its average accuracy rate for a given condition fell above (significantly better performance), below (significantly worse performance), or within (no significant difference) the confidence intervals of the model estimates per condition for each of the human groups. The full model results are presented in supplementary materials (Supplementary Table 3).

The model results confirm that the storage capacity of children have yet to reach adult-like levels (i.e. no overlap between confidence intervals estimated for each group on Set Size 4, 0-Swap trials). Furthermore, the parrot’s storage capacity was comparable to that of human adults, being at ceiling on all 0-Swap trials, and was higher than the modeled confidence interval for children on Set Size 4, 0-Swap trials. The results of the point estimate analysis on 0-Swap trials suggest the adult group as the more suitable benchmark for cross-species comparison, given that manipulation accuracy cannot exceed initial storage performance. We now turn to the modeled measures of mental manipulation capacity (swap trials).

As can be seen on Fig. 2, the parrot’s performance exceeded that of human adults, or fell within the modeled range of adult performance, on 12 or the 14 trial types. Only on sets of 4 trials, with 3- or 4- swaps, did its performance fall below that of human adults. Children were not given 4-swap trials. The performance of the parrot on Set Size 4, 3-Swap Trials (62.5% correct) was above that of the children (upper modeled confidence interval, 60.8), but by 4-Swaps clearly had fallen to the level shown by children with 3-swaps. The outcome of this analysis is that across Set Sizes 2 through 4, and number of swaps from 0 through 4, a Grey parrot, with no training on this task except observing two humans perform 3 trials, performed overall comparably to adults.

The one exception to this generalization was that increasing manipulation load at the highest set size led the parrot’s performance to plummet below human adults’, approaching that of 6-to-8-year old children. What factors may restrict the parrot’s manipulation performance when the system is taxed in this way? First, these factors may not be related to fundamental cognitive capacities at all. The parrot had been given 3 or 4 trials in this experiment every day for many months, and sometimes, near the end, when he saw the 4-cup set up (prior to trial onset), he simply refused to engage. That is, motivation might explain the decline. Second, though storage related factors (e.g. temporal decay) seem to have comparable effects on birds’ and primates’ memory capacities^29^, such factors may interact with set size and number of swaps to produce differences in manipulation ability. Moreover, constraints in attentional capacities may have led to the relatively poor performance. Manipulating 4 items multiple times requires that items be constantly switched in and out of the focus of attention. Although previous work demonstrates that attentional shifts do not limit human accuracy in a similar Shell Game task^25^, it is possible that manipulation ability in Grey parrots is supported by separate mechanisms, and that attentional shifts may prove detrimental when updating. Finally, other factors directly relating to the manipulation operation (e.g. interference between representations, stability of representations, depletion of a manipulation resource, etc.) may account for the patterns observed.

To begin to arbitrate among these possibilities, we conducted an additional experiment with the parrot, presenting it with the most difficult trials that it had previously experienced: 4-items involving 4 swaps. This study commenced 11 months after the previous experiment ended, so his motivation to participate was revived. Here, we kept the overall manipulation load constant (4 swaps), while systematically varying the number of times that the tested item itself was manipulated (i.e. whether it moved 0 to 4 times). If non-cognitive factors (e.g. motivation), or storage related factors (e.g. rate of temporal decay) previously limited the parrot’s performance, accuracy rates should be equal across all conditions – whether the tested item moved all 4 times or none at all. In contrast, if attentional switching had limited the parrot’s manipulation performance, accuracy rates should be highest when the tested item was involved in all 4 swaps (tested item constantly within focus of attention), as opposed to when it was involved in fewer swaps. Lastly, if factors more directly related to executing manipulation performance had limited Griffin’s performance, accuracy should decrease as the number of movements in which the target is involved increases. Given that this study was conducted with a single participant, we offer only a descriptive analysis of the data. Nonetheless, the observed results were qualitatively most consistent with this last hypothesis. The parrot’s performance accuracy was not comparable across all conditions. Rather, its accuracy rates were highest when the tested item was manipulated 0 times (Obs. Mean = 100%) and overall decreased as a function of the number of times it was manipulated (75%, 75%, 33%, 58.3% across 1–4 movement participations, respectively). Though future work is required to identify the factors limiting the parrot’s manipulation ability, these results suggest that the behavioral limits exhibited in the previous task are not likely to have resulted from non-cognitive factors, storage related factors, or constraints in attentional switching.

Taken together, the results of the current study suggest that manipulation ability is not a uniquely human capacity, and that overall comparable signatures of manipulation limits can be found in human adults and at least one Grey parrot. Although we had access to only a single parrot, following generally accepted arguments^30^, we suggest that his reliable performance at a given level represents the potential capacity of the species. It is possible that similar ecological pressures experienced by humans and non-human animals may have led to the development of similar VWM architecture within species evolutionarily separated by over 300 million years^17^. In contrast, functionally homologous neural structures supporting memory in humans and birds may similarly point towards homologous evolution of manipulation abilities^11^. Whether the similarities in behavioral limits observed here reflect convergent or homologous evolutionary processes requires further investigation. However, the intuitive and engaging nature of the Shell Game paradigm provides researchers with a means to pursue this issue. Future work systematically probing manipulation capacities in non-human animals may provide insights into identifying when manipulation ability emerged, determining its neurocognitive architecture, and understanding how/if its capacity changed across evolution. Comparing manipulation performance across Grey parrots and other bird species may be a viable starting point. For example, Grey parrots, in general, are capable of using symbolic representations^17,22^, and Griffin’s referential production of approximately 30 English words and comprehension of at least 40, including those for color labels, has been fully documented^17,22,24^. Comparing Shell Game performance across this species, in both those that have and do not have the capacity for verbal symbolic representation, can help determine the formats of representation over which manipulation operations are performed: For example, might Griffin have used his knowledge of referential color labels to engage in a verbal rehearsal strategy, a process unavailable to Grey parrots without such training, and that we could eliminate from human subjects? Similarly, comparing manipulation performance with birds that demonstrate higher storage capacities (e.g. caching crows) can provide insight into whether storage and manipulation rely upon a shared VWM resource versus separate resources.

More broadly, our findings may also provide insights into the nature of human intelligence. The ability to manipulate information in VWM constitutes the “working” part of memory, supporting a variety of broader cognitive abilities^1^. “Updating” constitutes a particular type of manipulation that is distinct from other executive functions^31^ and most strongly correlates with human intelligence^32,33^. Assuming that parrots demonstrate mental manipulation capacities comparable to those of human adults raises the question of what factors differentiate human intelligence from those observed in non-human animals. Though further work is required to determine whether Griffin’s abilities generalize beyond this case study of manipulation, understanding why its performance deteriorates, relative to that of adults, on 3- and 4-swap trials when 4 items are initially stored in the Shell Game can help address this question.

Specifically, identifying the nature of errors underlying manipulation failures may prove instrumental towards identifying differences between human and parrot updating skills. For example, misbinding errors (incorrectly combining the features of one item with that of another) observed in this task could suggest that manipulation costs stem from interference between stored representations. Alternatively, forgetting errors or those related to increased imprecision may present durability of representations/reconsolidation processes as a limiting factor for manipulation. Furthermore, observing how these errors change as a function of manipulation load can help identify whether they stem from the depletion of a fixed capacity resource versus a stochastic process where the probability of observing a failure simply increases with each manipulation event. Cross-species comparisons in these manipulation errors may also provide insight into the nature of mechanisms that support manipulation in a variety of animals. Evolutionary approaches towards characterizing VWM manipulation may prove invaluable towards characterizing the architecture of this system, the format of its representations, and the nature of its limiting factors.

## Methods

### Participants

#### Adults

Twenty-one Harvard University undergraduate students (18- to 30-years old; 12 females) with normal or corrected-to-normal vision took part in the study in exchange for course credit. All adult participants were tested in the Vision Sciences Laboratory at Harvard. An additional 6 participants were excluded from analysis, based on self-reported use of mnemonic strategies (n = 3) or inattentiveness/looking away during trials (n = 3).

#### Children

Twenty-one children (seven 6-year-olds, seven 7-year-olds, and seven 8-year-olds; 10 females) recruited from the greater Cambridge/Boston area with normal or corrected-to-normal vision took part in the study. All children were tested in the Laboratory for Developmental Studies at Harvard University, and received their choice of either $10 or a toy of the same value, in exchange for participation. An additional 4 children participated in the study, but were excluded from analyses due to experimenter error during testing (n = 1) or verbalizing colors aloud during trials (n = 3).

#### Parrot

Griffin, a 22-year-old male Grey parrot (*Psittacus erithacus*), was tested at Harvard University and received raw cashew quarters in exchange for his participation. Griffin has been the subject of cognitive and communicative studies (e.g. object permanence, Piagetian developmental tasks, vocal labeling), since his acquisition from a breeder at 7.5 weeks of age. Food and water were always available.

### Procedures

All aspects of the experiments described here were approved by the Harvard Internal Review Board (IRB18–1703, IRB18-2007) and the Harvard Animal Experimental Protocol review (13-06-164-2). All methods were carried out in accordance with relevant guidelines and regulations. Informed consent was obtained from all adult participants. In the case of children, informed consent was obtained from a parent and/or legal guardian.

Both adults and children completed the experiment during a single experimental session that lasted approximately 2 hours. The parrot was tested over a period of ≈50 days, completing approximately 2–3 trials per day. Adults and the parrot completed a total of 120 trials [8 trials for each combination of set size (2–4 items) and number of swaps (0–4 swaps)]. The children completed a total of 36 trials [3 trials for each combination of set size (2–4 items) and number of swaps (0–3 swaps)]. Prior to the onset of each trial, the experimenter told the children a portion of a story (max 3 sentences) about “Charlie the Cheetah”. Charlie is a magical cheetah with colorful spots that somehow kept getting lost. On each trial, the children were asked if they would help Charlie find his spots.

Trials and trial order were identical across all individuals. Namely, trial order was scaffolded, such that individuals were first presented with 2 item trials containing 0 swaps, followed by 2 item trials containing 1–4 swaps (intermixed). This pattern (0 swaps followed by 1–4 swaps) was repeated for set sizes 3 and 4. Participants were allowed to take a break (unlimited time) at any point, but were explicitly asked if they wished to do so after every 8 trials.

Prior to testing, all groups of participants completed a training phase. Adults and children were given verbal instructions, after which they performed six practice trials: (e.g. Set Size 2–0 Swaps, Set Size 2–2 swaps, Set Size 3–3 Swaps). In contrast, the parrot did not perform any practice trials. Instead, it passively viewed two humans perform examples of the aforementioned trials.

During testing, each adult or child was seated across from the experimenter, with a table in between the two individuals. Griffin was perched on a T stand (used for several previous experiments), with a stool between him and the experimenter. Stimuli consisted of colored woolen pompons (2.54 cm in diameter), whose colors (green, blue, white, orange, yellow) were sampled without replacement. These items were presented on a felt-covered tray (~30 cm in diameter) located on the center of the table/stool. Viewing distance was unconstrained, but averaged 25 cm for adults and children, and about 30 cm for the parrot. All participants had overhead views of the display.

Overall procedures were nearly identically for all groups of participants. At the beginning of every trial, the experimenter placed the colored items on predetermined locations on the felt for approximately 2 seconds. Note, to ensure that Griffin was paying attention during this period, the experimenter lifted the tray in front of his face, moving it to both sides of his head so he could see from both eyes, and told him to “look!”. For all participants, the experimenter then covered the colored items simultaneously with opaque black cups (2 oz.). The cups remained stationary for 1 second. On 0-swap trials, memory for a cued item was tested immediately after. On all other swap trials, the experimenters simultaneously moved two cups (following parabolic trajectories) until the cups had swapped positions over a 2 second period. The cups remained stationary for an additional second, before the experimenter initiated the subsequent swap. To maximize temporal control, the experimenter wore an ear-piece (connected to a laptop) that marked when each of these events should occur. Once all swaps were performed, memory for one item was tested. During this test period, the experimenter pulled out an additional colored item (previously hidden under the table) that matched the identity of an item hidden under a cup. The participant was instructed to touch the cup under which they expected that color to be (using a finger for adults and kids, beak for the parrot). For every correct response, all groups of participants were told “Good job!” Moreover, the children were additionally allowed to place the color pompom on a sticker sheet (an enlarged picture of Charlie), and the parrot was given a quarter of a raw cashew.

Given that independent limits exist for processing visual vs. verbal information in working memory^1^, we sought to minimize the recruitment of verbal encoding strategies that could be used to represent stimuli in a non-visual format. Thus, evidence demonstrating employment of a verbal strategy was used as a criterion for excluding participants. To prevent adults from verbalizing color stimuli, each participant was given a two-digit verbal pair that they were instructed to rehearse aloud (at a rate of approximately 1 digit/sec) during the duration of each trial^27,28^. All adult participants complied with these instructions. A pilot experiment demonstrated the verbal load to be too taxing for children. As such, children in the current experiment were monitored for verbally rehearsing color identities, and were also subsequently asked if they were using any such strategies during the experiment. Those who were doing so (n = 3) were excluded from analyses. Though Griffin can referentially label colors, the qualitatively differential performance across conditions for the 4-item, 4 swaps experiment suggest that it is unlikely that his performance reflects a verbal rehearsal strategy; that issue, however, is subject to further study.

While the parrot was being tested, a second experimenter who was seated several feet away would stop the trial if Griffin had not attended to the swaps (e.g. looked away). If he had, a mistrial was declared (n = 7), and this trial was repeated randomly after 5 trials had been subsequently performed. Otherwise, the first experimenter touched all four cups briefly so that the parrot could not respond based on what cups had been most recently touched. During the testing period, the parrot was asked to “Match X!”, where X = color of the pompon. Griffin was then required to tap a cup; the experimenter watched the bird’s beak, not the cups. The first cup tapped was considered his choice. Note was made whether he dithered. Note that parrots cannot follow human eye gaze for the distances involved, so that cuing via eye gaze is not an issue. All aspects of the additional 4-item, 4 swap experiment (parrot only) were identical to those described above, with the exception that the parrot performed a total of 60 trials presented in randomly intermixed order (12 trials per condition of 0–4 number of participation conditions). Prior to the onset of this second experiment, the parrot completed 12 trials of the 4-item, 0 swap condition with perfect accuracy, where a dwell period was additionally enforced to ensure that trials were as long as those used in the 4-item, 4 swap experiment.

### Linear mixed-effects modeling

The linear mixed-effects model (LMEM) used in the point estimate analysis was performed using the lme4 package (Version 1.1–21; Bates *et al*., 2019) implemented in R Version 3.6.1 (R Core Team, 2019). Adults’ and children’s data were fit by a model including main effects for set size and number of swaps, as well as the interaction of these two variables. Participant and trial variables were held as random effects. The model was run using the following code in R:

Model < − glmer(Response ~ Set_Size + Num_Swaps + Group + (1|Participant) + (1 | Trial), data = AllGroupsData, family = binomial, control = glmerControl(optimizer = c(“optimx”), optCtrl = list(method = “nlminb”)))

## Supplementary information

Supplementary Information.

## Data Availability

The datasets generated during and/or analyzed during the current study are available from the corresponding author on reasonable request.
