# Supplementary material for: Age and Species Comparisons of Visual Mental Manipulation Ability as Evidence for its Development and Evolution
Source: Sci Rep. 2020 May 6;10:7689. doi: 10.1038/s41598-020-64666-1 (PMC7203154; doi:10.1038/s41598-020-64666-1)
Supplement: Supplementary file 1 — Supplementary Information. [file 41598_2020_64666_MOESM1_ESM.pdf]

SUPPLEMENTARY INFORMATION

Age and Species Comparisons of Visual Mental Manipulation Ability as Evidence for its Development and Evolution

*Hrag Pailian
Harvard University

Susan E. Carey
Harvard University

Justin Halberda
Johns Hopkins University

Irene M. Pepperberg
Harvard University

Supplementary Experiment 1: Computerized Variant of Shell Game (Adults Only)

To ensure that the behavioral pattern observed in the main experiment cannot be attributed to either temporal decay or strategy use, we conducted a computerized variant of the Shell Game with human adults. Once more, participants were presented with 2-4 colors that swapped 0-4 times. However, in this experiment, a dwell period was enforced for each condition after all swaps were completed, such that the overall trial duration was held constant across all conditions (i.e. a 0 swap trial lasted just as long as a 4 swap trial). Thus, observed behavioral limits in manipulation ability could not be attributed to factors relating to temporal decay. Moreover, to prevent participants from using an "n-1" tracking strategy (attending to fewer items in the display and inferring the identity of an untracked item), we altered the way in which the target was tested. Here, a probe appeared around one of the items, and participants were instructed to identify the color of that target by choosing from a color bar of 8 possible color options.

Participants

Thirteen undergraduates from the Johns Hopkins University with normal or corrected-to-normal vision took part in the study in exchange for course credit. All experiments described in this study were approved by the school's internal review board. Sample size was chosen based on a similar pilot experiment containing the same number of participants.

Equipment

The experiment was conducted in a dimly lit room. Stimuli were presented on a Macintosh iMac computer with a viewable area of 43.5 x 27 cm. Viewing distance was not fixed, but averaged approximately 57 cm.

Stimuli

Prior to the onset of each trial, participants were presented with a two-digit (each measuring 1.7° x 0.85° of visual angle) verbal load at the center of the screen. All memory displays consisted of colored circles (diameter of each circle: 1.23° of visual angle), whose locations were randomly chosen from four vertices of an imaginary square (7.35° x 7.35° of visual angle) that was located at the center of the screen. The colors with which these circles could appear were randomly chosen without replacement from eight discrete categories: red, cyan, yellow, green, blue, orange, brown, and magenta. Each circle was framed by a circular white outline, whose thickness measured 0.13° of visual angle.

Procedure

Participants completed 300 randomly shuffled trials of a computerized Shell Game task (Supplementary Figure 1). The start of each trial was marked by a central fixation cross (black, 0.5° x 0.5° of visual angle) that was presented for 500 ms. After an interstimulus interval of 100 ms, participants were presented with a two-digit verbal load that they were previously instructed to rehearse out loud (≈1 digit/sec) throughout the trial. An experimenter was seated behind the participant throughout the duration of the entire trial, to ensure compliance with these instructions. These digits (Font Size: 35, Font: Calibri, Color: white) were presented on the screen for 500 ms, and were followed by a memory display, after an interstimulus interval of 1000 ms. Each memory display consisted of a varying set size of colored circles with white outlines. This display was presented for 500 ms, and was followed by a screen in which the colors of the circles disappeared, leaving behind the white outlines. After the colors disappeared, the display remained static for a memory consolidation period lasting 1900 ms. Following this consolidation period, one of two trial types occurred (i.e., no movement or movement trials). In the no movement condition (0 swaps), the consolidation period was

immediately succeeded by the presentation of the test display. In this way, performance on 0 swap trials was used to estimate limitations in storage capacity.

In the movement conditions, the consolidation period was followed by dynamic displays where two of the circular items (still defined by their white outlines) proceeded to swap positions at a rate of 100 pixels per frame. During each swap, the pair of targets would move smoothly across the screen, following a parabolic trajectory, passing each other, and then come to rest in their new positions. Dynamic trials included 1, 2, 3, or 4 swaps. Each swap lasted an average of 1650 ms, and was separated by a 1600 ms reconsolidation period prior to the onset of the next event (i.e., either the test display or another swap). The selection of which targets would participate in a given swap was random and unconstrained. The observer's task was to watch the swap and attempt to update their representations of where each color would appear in the test display. Thus, performance on movement trials was used to estimate the observer's ability to dynamically update color-location information in visual working memory.

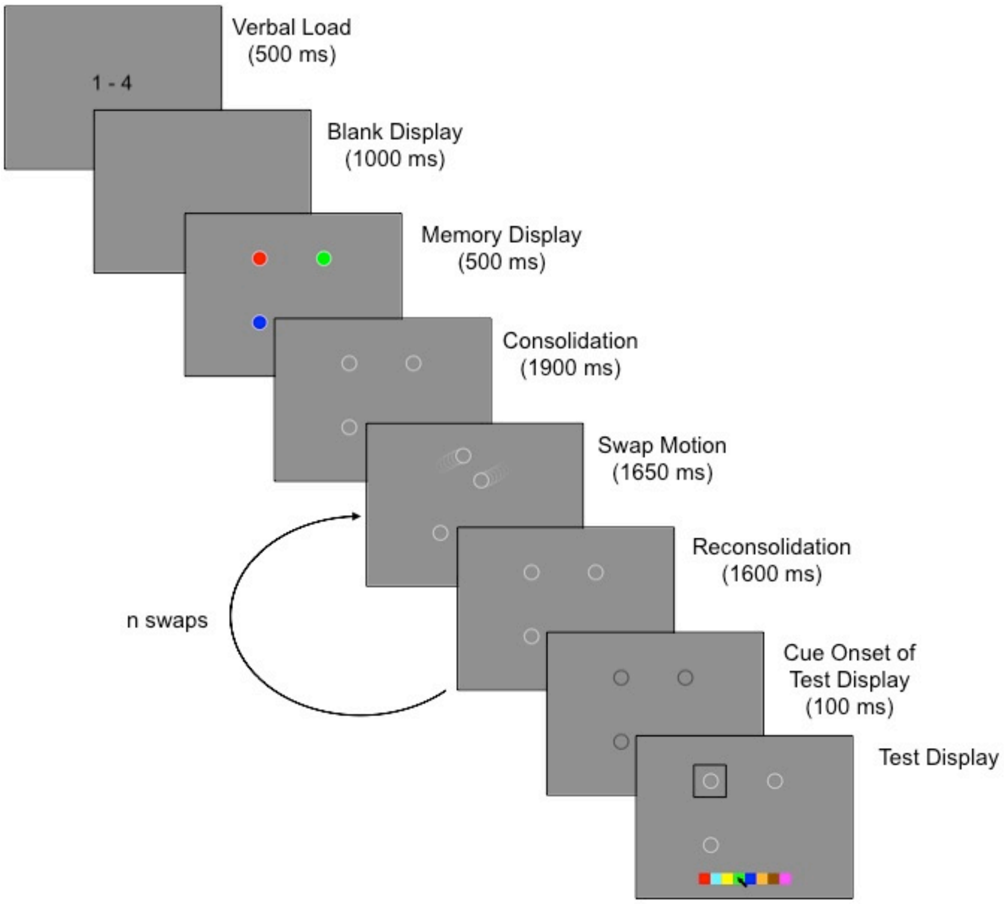

Supplementary Figure 1. Schematic of typical trial in computerized variant of Shell Game.

After all swaps and consolidation periods were completed, all items remained stationary for a variable period of time, such that overall duration was held constant across all conditions (≈16.9 seconds). This dwell period was subsequently followed by the test display. In both no movement and movement conditions, a black square outline (2.45° by 2.45° of visual angle) appeared around one of the items. Participants had to indicate the identity of that stimulus by using the mouse to click on a color that appeared in a color bar. The color bar was always comprised of 8 squares that were arranged horizontally and contained all of the possible discrete colors used in the memory display. This method provides the additional advantage of characterizing the types of errors participants make in this task (reporting the identity of a color presented in the memory display vs. one that was not shown at all). By changing the response mode in this way, participants were encouraged to encode the identities of all items.

Results:

Results are illustrated in Supplementary Figure 2. A 3 (set size: 2-4) x 5 (number of swaps: 0-4) within-subjects ANOVA revealed a significant main effect of set size, $F(2,46)=40.09$, $p<.001$. Performance was highest for Set Size 2, which differed from both Set Size 3, $F(1,46)=28.98$, $p<.05$, and Set Size 4, $F(1,46)=61.29$, $p<.05$. Performance for Set Size 3 was also significantly better than Set Size 4, $F(1,46)=21.24$, $p<.05$. Furthermore, the ANOVA revealed a significant main effect of number of swaps $F(4,92)=10.11$, $p<.001$, as performance decreased with increasing numbers of swaps. Specifically, performance was higher in the static (0 swaps) condition compared to the dynamic (1-4 swaps) conditions, $F(1,92)=34.84$, $p<.05$, suggesting that there is an increased cost associated with manipulating representations, relative to simply storing them in visual WM. The ANOVA also revealed a significant interaction of set size x number of swaps, $F(8,184)=2.64$, $p<.03$. A significant linear (Set Size 2) x linear (Set Sizes 3 and 4) contrast was observed, $F(1,184)=22.79$, $p<.05$, suggesting differential effects for manipulating 2 items in visual WM, compared to manipulating 3 or 4. Taken together, these results demonstrate little to no cost for manipulating 2 items, whereas performance systematically decreases as a function of the number of swaps when manipulating 3 or 4. These results replicate the pattern observed on the main experiment, while controlling for factors relating to temporal decay and n-1 tracking strategies.

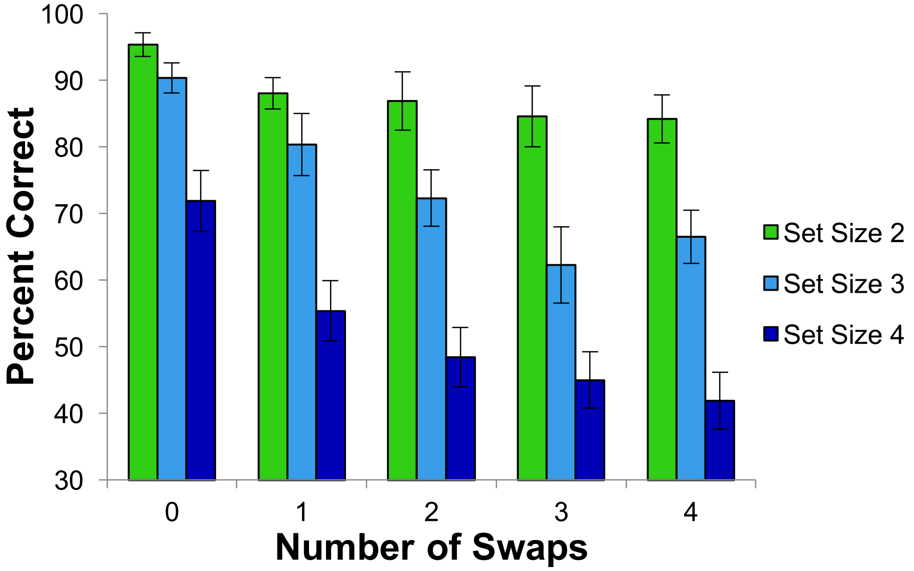

Supplementary Figure 2. Performance accuracy for each set size by number of swaps condition in a computerize variant of the Shell Game. Pattern of results observed here replicate those in the live version mentioned in the main body of the current investigation.

Full Report of Three-Way ANOVA

Within Subjects Factors: **Set Size** (2-4) and **Number of Swaps** (0-3)
Between Subjects Factor: **Group** (Adults vs. Children)

Supplementary Table 1. Full report of three-way ANOVA

	WITHIN SUBJECTS FACTORS				
	**Sum of Squares**	**Degrees of Freedom**	**Mean Square**	**F**	**Sig.**
**Set_Size**	37807.54	2	19645.51	53.84	0.00
**Set_Size * Group**	3482.14	2	1809.39	4.96	0.01
**Error(Set_Size)**	28088.21	80	364.88		
**Num_Swaps**	19184.41	3	7441.41	17.59	0.00
**Num_Swaps * Group**	2616.95	3	1015.08	2.40	0.08
**Error(Num_Swaps)**	43634.12	120	423.13		
**Set_Size * Num_Swaps**	6979.72	6	1553.45	4.14	0.00
**Set_Size * Num_Swaps * Group**	709.88	6	157.99	0.42	0.81
**Error(Set_Size * Num_Swaps * Group)**	67365.38	240	374.83		
	BETWEEN SUBJECTS FACTOR				
	**Sum of Squares**	**Degrees of Freedom**	**Mean Square**	**F**	**Sig.**
**Intercept**	3581721.54	1	3581721.54	2858.03	0.00
**Group**	26979.48	1	26979.48	21.53	0.00
**Error**	50128.55	40	1253.21		

Bootstrapping Parrot's Performance

To investigate whether the parrot's performance on each condition significantly differed from chance (50%, 33.33% and 25% for Set Sizes 2-4, respectively), we performed a bootstrap analysis. For each condition of set size by number of swaps, we randomly sampled 8 trials (with replacement) from the observed data and calculated the mean of this sample. By repeating this procedure across 10,000 iterations, we obtained a grand mean across all these samples and 95% confidence intervals. The results of this bootstrap analysis suggest that the parrot's performance across all conditions exceeded chance performance for that set size, as the 95% confidence intervals did not overlap with chance values. Supplementary Table 2 presents the means of the 10,000 iterations for each condition, along with confidence intervals and chance level for that condition. The distribution of means across the 10,000 iterations are presented in Supplementary Figure 3.

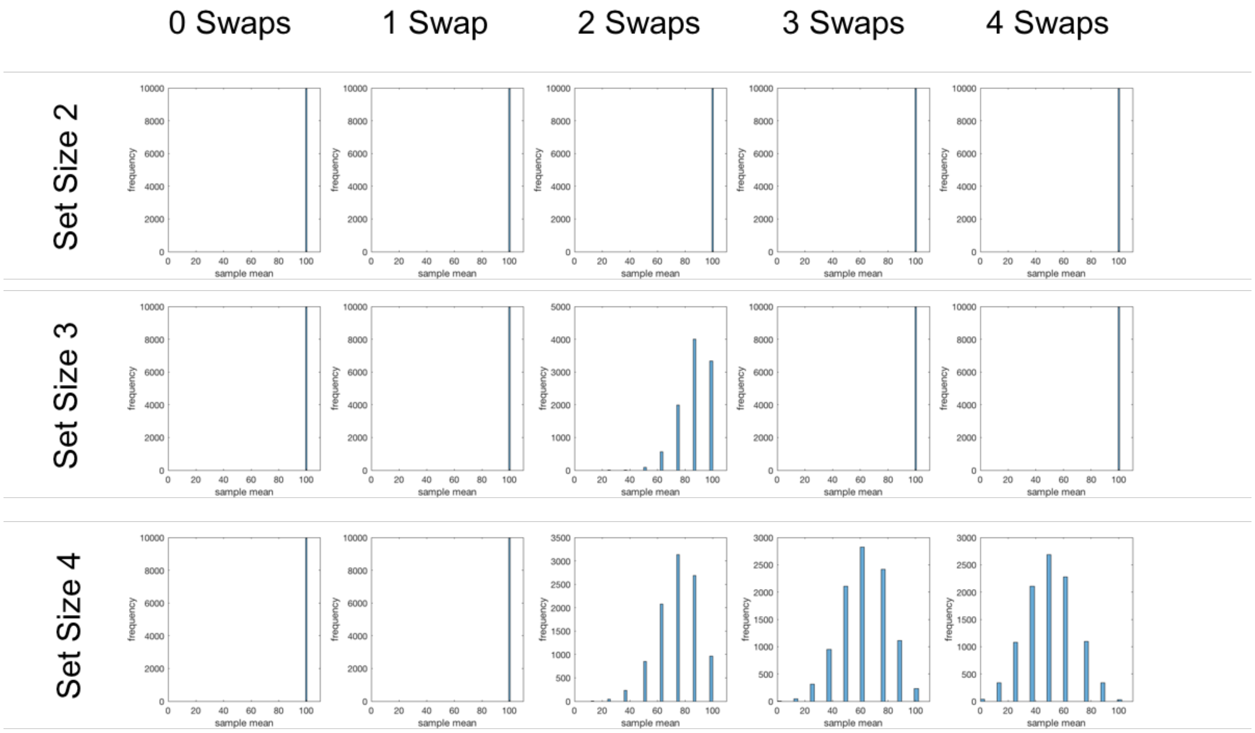

Supplementary Figure 3. Frequency distribution of Griffin's bootstrapped results (for each combination of set size by number of swaps) observed across 10,000 iterations (sampled with replacement)

Bootstrapping Parrot's Performance

Set Size	Num Swaps	Mean	Lower C.I.	Upper C.I.	Chance
2	0	100.00	100.00	100.00	50.00
2	1	100.00	100.00	100.00	50.00
2	2	100.00	100.00	100.00	50.00
2	3	100.00	100.00	100.00	50.00
2	4	100.00	100.00	100.00	50.00

3	0	100.00	100.00	100.00	33.33
3	1	100.00	100.00	100.00	33.33
3	2	87.40	87.17	87.63	33.33
3	3	100.00	100.00	100.00	33.33
3	4	100.00	100.00	100.00	33.33
4	0	100.00	100.00	100.00	25.00
4	1	100.00	100.00	100.00	25.00
4	2	75.05	74.75	75.35	25.00
4	3	62.17	61.83	62.51	25.00
4	4	49.88	49.53	50.23	25.00

Supplementary Table 2. Griffin's bootstrapped performance for each set size by number of swaps condition. Means, as well as lower and upper 95% confidence intervals), calculated across 10,000 iterations. Chance levels for each condition provided for comparison.

Cross-Species Comparisons: Results of Generalized-Linear Mixed Effects Model

ADULTS						
Set Size	Num Swaps	Model Estimate	Lower C.I.	Upper C.I.	Parrot Accuracy	Griffin Compared to Modeled Data
2	0	99.2	97.8	99.7	100	Better
	1	98.3	96.2	99.2	100	Better
	2	97.2	94.6	98.5	100	Better
	3	99.1	97.6	99.6	100	Better
	4	99.0	96.1	99.8	100	Better
3	0	98.9	97.2	99.5	100	Better
	1	94.8	91.0	97.0	100	Better
	2	90.7	85.2	94.3	87.5	Equal
	3	90.5	84.9	94.1	100	Better
	4	78.9	69.2	86.2	100	Better
4	0	96.6	93.6	98.2	100	Better
	1	88.3	82.0	92.6	100	Better
	2	80.0	71.3	86.5	75	Equal
	3	79.1	70.2	85.9	62.5	Worse
	4	72.2	61.4	80.9	50	Worse

CHILDREN (6-to-8-Year Old's)						
Set Size	Num Swaps	Model Estimate	Lower C.I.	Upper C.I.	Parrot Accuracy	Griffin Compared to Modeled Data
2	0	97.0	91.6	98.9	100	Better
	1	93.3	86.5	96.8	100	Better
	2	89.5	81.4	94.3	100	Better
	3	96.3	90.9	98.6	100	Better
3	0	95.6	89.8	98.2	100	Better
	1	81.9	71.6	89.0	100	Better
	2	70.8	58.6	80.5	87.5	Better
	3	70.2	58.0	80.0	100	Better
4	0	87.4	78.6	93.0	100	Better
	1	65.2	52.7	76.0	100	Better
	2	49.8	37.6	62.0	75	Better
	3	48.5	36.4	60.8	62.5	Better

Supplementary Table 3. Estimated means (including lower and upper 95% confidence intervals) produced by the generalized linear mixed effects model. Cross-species comparisons

conducted by determining whether the parrot's average accuracy for a given condition fell above (significantly better performance), below (significantly worse performance), or within (equal) these lower and upper bounds.
